# Nitrogen Fixing *Azotobacter* Species as Potential Soil Biological Enhancers for Crop Nutrition and Yield Stability

**DOI:** 10.3389/fmicb.2021.628379

**Published:** 2021-02-25

**Authors:** Abderrahim Aasfar, Adnane Bargaz, Kaoutar Yaakoubi, Abderraouf Hilali, Iman Bennis, Youssef Zeroual, Issam Meftah Kadmiri

**Affiliations:** ^1^Green Biotechnology Laboratory, Moroccan Foundation for Advanced Science, Innovation and Research (MAScIR), Rabat Design Centre, Rabat, Morocco; ^2^Laboratory of Health Sciences and Technologies, High Institute of Health Sciences, Hassan 1st University, Settat, Morocco; ^3^AgroBioSciences–Microbiome, Laboratory of Plant-Microbe Interactions, Mohammed VI Polytechnic University, Ben Guerir, Morocco; ^4^Situation Innovation Group–OCP Group, Jorf Lasfar, Morocco

**Keywords:** *Azotobacter*, biological nitrogen fixation, nitrogenase, nitrogen, plant nutrition, phosphate

## Abstract

Biological nitrogen fixation (BNF) refers to a microbial mediated process based upon an enzymatic “Nitrogenase” conversion of atmospheric nitrogen (N_2_) into ammonium readily absorbable by roots. N_2_-fixing microorganisms collectively termed as “diazotrophs” are able to fix biologically N_2_ in association with plant roots. Specifically, the symbiotic rhizobacteria induce structural and physiological modifications of bacterial cells and plant roots into specialized structures called nodules. Other N_2_-fixing bacteria are free-living fixers that are highly diverse and globally widespread in cropland. They represent key natural source of nitrogen (N) in natural and agricultural ecosystems lacking symbiotic N fixation (SNF). In this review, the importance of *Azotobacter* species was highlighted as both important free-living N_2_-fixing bacteria and potential bacterial biofertilizer with proven efficacy for plant nutrition and biological soil fertility. In addition, we described *Azotobacter* beneficial plant promoting traits (e.g., nutrient use efficiency, protection against phytopathogens, phytohormone biosynthesis, etc.). We shed light also on the agronomic features of *Azotobacter* that are likely an effective component of integrated plant nutrition strategy, which contributes positively to sustainable agricultural production. We pointed out *Azotobacter* based-biofertilizers, which possess unique characteristics such as cyst formation conferring resistance to environmental stresses. Such beneficial traits can be explored profoundly for the utmost aim to research and develop specific formulations based on inoculant *Azotobacter* cysts. Furthermore, *Azotobacter* species still need to be wisely exploited in order to address specific agricultural challenges (e.g., nutrient deficiencies, biotic and abiotic constraints) taking into consideration several variables including their biological functions, synergies and multi-trophic interactions, and biogeography and abundance distribution.

## Introduction

Intensive agriculture relies on important application of N fertilizers, along with other essential nutrients for maximizing crop productivity. Generally, application of synthetic N-based fertilizers was estimated to produce approximately half of the global food supply and that consumption rate of N fertilizers is projected to trend upward from 80 to 180 Mt by 2050 (Bindraban et al., 2015). On the other hand, up to 50% of the application of conventional N-based fertilizers is subject to loss into the soil and the environment (Singh et al., 2014, 2015a). This could substantially inflict economic and environmental issues such as increasing greenhouse gas emissions (e.g., nitrous oxides volatilization accounts for approximately 10-fold emission of CO_2_-equivalent), soil acidification, depletion of non-renewable resources and nitrate leaching into the groundwater and surface water, which can cause devastating effects such as water eutrophication. Thus, there is a need to sustain use of N fertilizers in order to meet agriculture sustainability challenges consisting of a better crop nutrition and productivity needed for the ever-increasing world population. Most importantly, the soil ecosystem services with safe provision are, undoubtedly, a must for securing agroecosystems sustainability (Lescourret et al., 2015).

Meeting such an urgent and rapidly increasing demand for food, notably in developing nations, cannot be achieved without appropriate mineral fertilization supplies and best practices, especially where crops and resources hardly contribute to an efficient crop production. In fact, constant efforts are needed to intensify agricultural production in a sustainable manner, which consider the entire agro-ecosystem, bio-chemical diversity with the potential to mitigate the adverse impacts of low soil fertility, abiotic stresses, pathogens, and pests (Tilman et al., 2011; Bargaz et al., 2018).

In this context, there is a growing need to consider new innovative approaches for smart and sustainable “food and feed” production with a lesser reliance on conventional fertilizers, notably N. This is in line with meeting current and future changes in human needs within a sustainable context that will likely depend on best management practices and wiser exploitation of both biological and mineral resources, while maintaining environmental quality including preserving natural resources. However, securing adequate plant N nutrition for such a highly mobile nutrient in soils remains challenging. In this regard, biologically fixed N has been the major input of N in agroecosystems.

Atmospheric nitrogen (N_2_)-fixing bacteria inhabit both plant tissues (e.g., nodules, roots) and soil-root rhizosphere interface and can, consequently, supply significant N amounts for plant growth. This is mainly due to the “biological nitrogen fixation (BNF)” microbially mediated process through a highly sensitive bacterial enzymatic conversion of atmospheric N_2_ into ammonia (NH_3_). BNF can provide an ecologically acceptable complement or substitute for mineral N fertilizers. This process is controlled by the availability of some important resources such as phosphate (P), Molybdenum (Mo), and water (Schulze and Drevon, 2005; Alkama et al., 2012; Lazali and Bargaz, 2017; Timmusk et al., 2017). Published estimates regarding N derived from BNF indicated rates ranging approximately between 1.95 × 10^11^ kg of N-NH_3_ (Galloway et al., 2004) and 2.5 × 10^11^ kg of N-NH_3_ from BNF is fixed annually (Cheng, 2008). This study also reported that nearly 2 tons of industrially fixed N is needed as fertilizers for crop production to equal the effects of 1 ton of biologically fixed N by leguminous crops and cyanobacteria.

The process of BNF is widely known as (i) symbiotic N fixation (SNF) by bacteria living in symbiotic association with leguminous and higher plants that allocate carbon to N_2_-fixing bacteria in exchange for N and (ii) non-symbiotic BNF by heterotrophic or autotrophic bacteria inhabiting soils, water, rocks, and leaf litter or in association with plants. For example, rhizobia–legume symbiotic associations are known to be the most important BNF biosystem, contributing with an average of 227 kg N ha^–1^ annually (Herridge et al., 2008) and may reach up to 300 kg N ha^–1^ according to Roughley et al. (1995). Meanwhile, non-symbiotic BNF estimates for maize, rice, and wheat production systems reported an average contribution of 15.5 kg N ha^–1^ based on a 50-year assessment study (Ladha et al., 2016).

Large-scale data on non-symbiotic BNF estimates are scarce except for staple cereal crops such as maize, rice, and wheat (Ladha et al., 2016). However few reports indicated that BNF by free-living diazotrophs may be roughly estimated at up to 60 kg N ha^–1^ year^–1^ (Vadakattu et al., 2006; Reed et al., 2011). Among non-symbiotic N_2_-fixing bacteria; *Beijerinckia*, *Azotobacter*, *Azospirillum*, *Herbaspirillum*, *Gluconacetobacter*, *Burkholderia*, *Clostridium*, *Methanosarcina*, and *Paenibacillus* are well-known and have proven significant efficacy in cereals crops (e.g., growth and grain yield) (Malik et al., 2002; Kennedy et al., 2004; Ritika and Utpal, 2014; Ladha et al., 2016). In this review, we focused on *Azotobacter* being non-symbiotic N_2_-fixing bacteria that are highly diverse and globally widespread in soils. This bacterial group may represent the dominant natural source of N in ecosystems lacking SNF (Choudhury and Kennedy, 2004; Das and Saha, 2007). Moreover, the abundance of *Azotobacter* species in the soil could improve the availability not only of N through the BNF processes (Din et al., 2019), but also P as well (Velmourougane et al., 2019). Moreover, a study by Kizilkaya (2009) demonstrated that soil carbon and sulphur contents increased in response to inoculation with *Azotobacter* species by accelerating the mineralization of soil organic residues, which subsequently reduced heavy metals absorption by roots.

Recently, advancing applied research on *Azotobacter* species is of special interest as both agriculturally important plant growth promoting N_2_-Fixing rhizobacterium (PGPR) that can be used for improving plant N nutrition and a biofertilizer based products at large scale, having significant improvements in crop productivity and soil fertility. Besides BNF, *Azotobacter* species are able to influence directly plant growth by synthesizing plant growth hormones [e.g., Indole Acetic Acid (IAA), gibberellins, and cytokinins]. These hormones can not only enhance plant growth and nutrient uptake, but can also indirectly protect host plants from phytopathogens and stimulate other beneficial rhizosphere microorganisms (Sahoo et al., 2014; Arora et al., 2018). Furthermore, *Azotobacter* strains exhibited positive effects on plant growth, crop yield and plant N requirements of several economically important cereal and pulse crops, reaching significant yield improvement (up to 40%) (Yanni and El-Fattah, 1999; Choudhury and Kennedy, 2004; Kannan and Ponmurugan, 2010; Wani and Ali, 2013; Ritika and Utpal, 2014). These positive traits offer promising possibilities to ecologically engineer *Azotobacter* species likely providing significant N inputs, while reducing reliance to N-containing fertilizers such as urea (Wani et al., 2016; Bageshwar et al., 2017).

Bioformulation of microbial inoculants is still requiring fundamental and applied studies, allowing the transition to a larger scale supporting the approach “from industry to farm” (Bashan et al., 2014). *Azotobacter* species possess some unique features such as cysts formation confering resistance to environmental stresses (Sadoff, 1975). Such properties are reviewed with potentialities to develop specific *Azotobacter* cyst-based formulations. Furthermore, besides the agronomic potential of *Azotobacter* based biofertilizers species, their geographical distribution and diversity require additional specific studies even though global interest in beneficial *Azotobacter* species has slightly waned in recent decades with a few thousands of research investigations on free N_2_-Fixing bacteria and *Azotobacter* in particular. Particularly, this review provides necessary data on *Azotobacter* species occurrence in Moroccan soils. This review also sheds light on specific *Azotobacter* features that are highly beneficial for improved crop production, nutrient use efficiency (particularly P and N), and stress tolerance in staple crops while highlighting their abilities to reduce the need for synthetic N-based fertilizers. Another part of this review summarizes patents related to *Azotobacter* formulations and product development bringing to light future prospects toward *Azotobacter* product innovation. Market aspects of *Azotobacter* based products are also discussed, allowing an evaluation of investments and the inventiveness in this field.

## *Azotobacter*: An Upward Trend Publication Rate of a Multifaceted Rhizo-Bacterium

The commercial history of microbial biofertilizers was launched with the Rhizobium-based bioinoculant named “Nitrogin,” which was considered the pioneer biofertilizer of all rhizobial inoculants (Patil and Solanki, 2016). Exploring the plant growth promoting abilities of soil N_2_-fixing microorganisms (including non-symbiotic bacteria such as *Azotobacter*) led to the development of the *Azotobacter*-based biofertilizer namely “azotobakterin” in Russia and East European countries, where ≃10 million ha of land was treated with microbial formulations in the middle of 19th century (Brown, 1974; Rovira, 1991). In Africa, the first studies on *Azotobacter* dated back to 1959, mainly reported by Becking, specifically on the genus *Beijerinckia* in South African soils (Becking, 1959). Few other studies published later by Makawi (1973) and Hegazi (1979) regarding the presence of *Azotobacter* species in Libyan and Egyptian soils (in both soils and roots).

**FIGURE 1:**
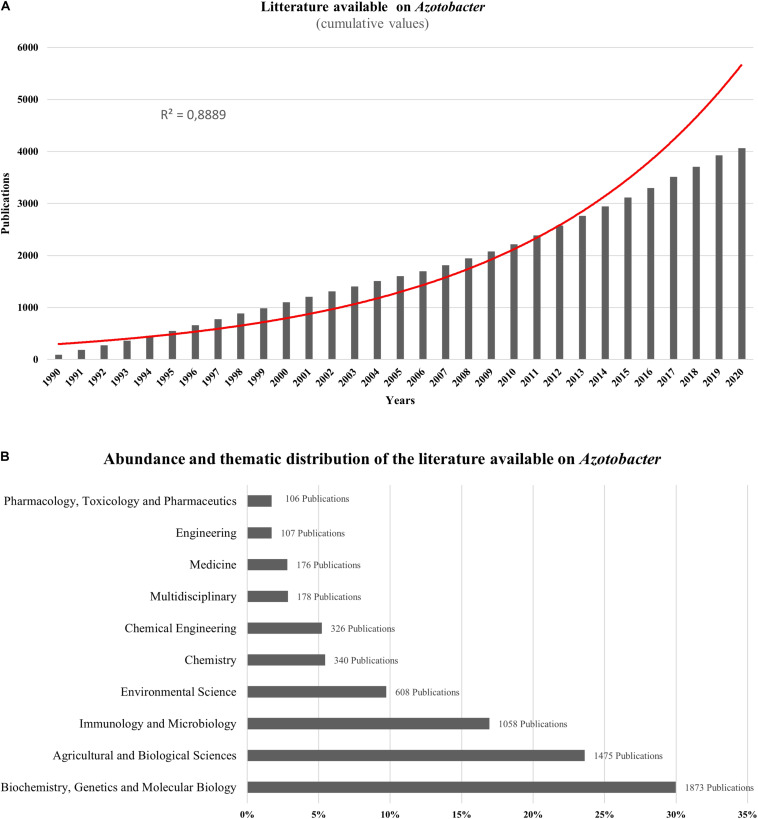
**(A)** Exponential growth in the number of scientific publications related to *Azotobacter* from 1990 to 2020 using *Azotobacter* as a key word from scopus (https://www.scopus.com). **(B)** Abundance and thematic distribution of the literature available on *Azotobacter*. Bibliometric research made in scopus (https://www.scopus.com) using *Azotobacter* as a keyword in June 2020.

*Azotobacter*-related research papers account for more than 4000 publications over the last two decades showing an exponential increase in the cumulative publication number, particularly from 1990 to 2020 reaching almost 4066 documents in 2020 found online using *Azotobacter* as a main key word (Figure 1A). As per research domain, most publications available on *Azotobacter* species were split between more than 20 research areas with most studies focused on biochemistry, genetics, molecular biology, agriculture, and overall biological sciences of *Azotobacter* (Figure 1B). Generally, *Azotobacter*-related publications are primarily research articles (88.7%) with more than 3600 publications, while reviews, conference papers and book chapters represent nearly 11.3%. As per country, the spatial distribution of *Azotobacter*-related documents revealed India and United States as the leading countries with more than 800 published items, whereas, research publications across African countries remain scarce, with the exception of Egypt wherein approximately 109 documents published between 1990 and 2020.

## Biogeographical Distribution and Functional Diversity of Azotobacter Species: (Case Study: Moroccan Soils)

*Azotobacter* representatives can commonly be found in soil, water, sediments, and plant roots (Aquilanti et al., 2004). *Azotobacter* species are generally found in slightly acidic to alkaline soils, which often governs the occurrence of certain species (Becking, 2006). For example, species belonging to *Azotobacter chroococcum* and *Azotobacter vinelandii* are more abundant in tropical soils, while *Azotobacter beijerinckii* species were often reported in acidic soils (Kennedy et al., 2015). However, only *Azotobacter paspali* was described to specifically associate with plant roots of *Paspalum notatum cv Batatais* (Kennedy et al., 2004).

The number of *Azotobacter* strains in soils is generally low (<10^4^ CFU g^–1^ soil). However, they are found throughout the world typically in 30 to 80% of sampled soils (Kennedy et al., 2004). Considerations about whether *Azotobacter* is a rhizospheric or non-rhizospheric bacterium are still debated. However, based on most research investigations, *Azotobacter* prevalence is generally not higher in the rhizosphere compared to open locations (Bartholomew, 2015). Nevertheless, certain species are denser in the rhizosphere of higher plants than in the soil itself. This is in line with the fact that *Azotobacter* species were reported to be found in fertile than in sandy soils owing to their relatively high requirement for P (Brenner et al., 2005). Likewise, findings regarding the ability of *Azotobacter* species to enhance the growth of various crops should prompt a re-examination of whether *Azotobacter* abundance might be higher in the rhizosphere than in non-rhizosphere soils (Kennedy et al., 2004).

Progress in culture-independent approaches, mainly shotgun and amplicon sequencing, could improve substantially our understanding of diversity and function of diazotrophs including *Azotobacter* species in soils and plant compartments. Recently, Hassen et al. (2020) reported low abundance of *Azotobacter* (0.06%) in the rhizosphere microbiome of a South African indigenous legume “*Cyclopia intermedia*,” as revealed by shotgun metagenomics techniques. Microbiome analysis of Maize rhizosphere in Pakistan based on library constructions of 16S rRNA and functional *nif-H* gene revealed biases linked to culture media in the culture dependent techniques to investigate relative abundance of diazotrophs in the rhizosphere (Qaisrani et al., 2019). *Nif-H* gene clones confirmed a relatively low abundance of *Azotobacter* (5%) among the diazotrophs investigated in this study (Qaisrani et al., 2019).

According to Rana et al. (2020) there is no strong evidence that *Azotobacter* members could colonize internally plant tissues, even if endophytic microbes are, theoretically, able to fix more N_2_ as compared to rhizospheric microorganisms because of low partial oxygen pressure in tissues compared to external surrounding soil.

**FIGURE 2:**
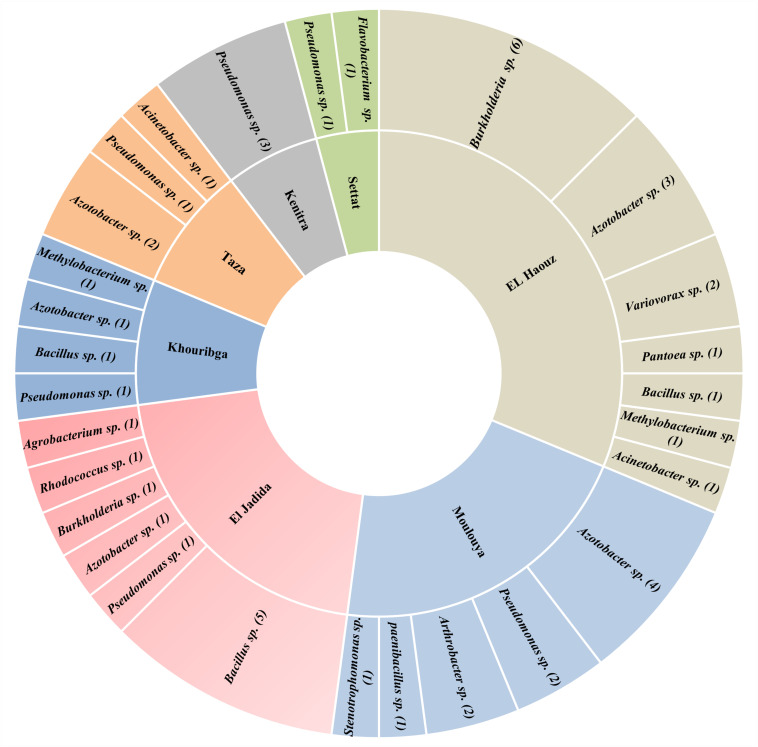
Number and distribution of major free N_2_-Fixing species identified in seven agricultural locations representative of Moroccan agricultural soils using 16S rDNA marker.

Meanwhile, either biogeographically or functionally, *Azotobacter* species have rarely been thoroughly investigated, mainly in Africa. Yet very few researches described the diversity and occurrence of *Azotobacter* species, which means that tremendous efforts are still to be deployed for mechanistic studies. In North Africa, only one main study was reported in Morocco by Sasson and Daste (1961), who published observations concerning *Azotobacter* ecology in dry soils of Morocco so far being the first evidence of the presence of *Azotobacter* occurrence strains in Moroccan soils. However, in a recent collaborative project aiming on isolation of *Azotobacter* from Moroccan soils will bring to light new evidence on the occurrence and diversity of *Azotobacter* and diazotrophs in Morocco (not published). Authors of this project (Dr. Issam Kadmiri. personal communication) adopted a molecular approach based on 16S rDNA and *nif-H* markers along with a conventional culture dependant approach and biochemical characterization. Promising findings showed significant variations in terms of both abundance and diversity of non-symbiotic N_2_-fixing isolates (Figure 2) in which, *Azotobacter* spp. representatives accounted for more than 22% of all strains isolated across Moroccan soils.

## Nitrogen Fixation, Plant Growth Promoting Traits and Stress Tolerance of *Azotobacter* Species

Despite various experimental data available on *Azotobacter* biostimulation traits on overall plant growth, the exact mode of action by which *Azotobacter* can enhance plant growth is not yet fully understood (Sumbul et al., 2020). However, the main mode of action evidently includes BNF, considering the capacity of these bacteria to fix N_2_, a vital macronutrient for plant growth. Moreover, these diazotrophs are capable to solubilize insoluble P forms in the soil (Nosrati et al., 2014). Other studies proposed other modes of action such as the production of phytohormone-like substances that alter plant growth and morphology and the bacterial mechanism of nitrate reduction that increases N accumulation in plants inoculated with *Azotobacter* strains (Deubel and Merbach, 2005; Wani and Ali, 2013; Wani et al., 2016).

### Nitrogen Fixation by *Azotobacter* Species

*Azotobacter* species play an important role in maintaining soil N status. The estimated contribution of non-symbiotic BNF rates are subject to variations due to several factors including environmental variability, management and cropping practices, genotypic differences, and technical aspects related to methods used to estimate BNF (Peoples and Herridge, 2000; Ladha et al., 2016). The rates of these free-living N_2_-Fixing bacteria to N input of soil range from 0.3 to 15 kg ha^–1^ year^–1^ (Saha et al., 2017), other studies reported up to 60 kg ha^–1^ year^–1^ (Bhattacharyya and Jha, 2012).

This BNF process under aerobic conditions is the principal characteristic of the genus, which is extremely tolerant to oxygen during fixing N_2_ due to respiration protection of nitrogenase (Hakeem et al., 2017). The two component proteins of the Mo-dependent nitrogenase are called the iron (Fe) protein or dinitrogenase reductase. These two component proteins act together to catalyze the reduction of dinitrogen in a complex reaction with an ideal reaction stoichiometry shown as follows (1) (Kirn and Rees, 1992):

(1) $$\begin{array}{c} {\text{N}}_{2}+8{\text{e}}^{-}+16MgATP+8H^{+}\\ \to 2{\text{NH}}_{3}+{\text{H}}_{2}+16MgADP+16Pi \end{array}$$

**FIGURE 3:**
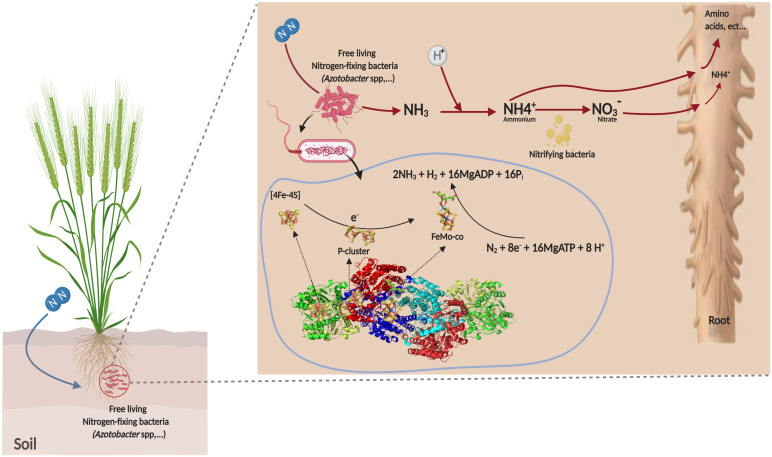
Mechanism of non-symbiotic fixation of atmospheric nitrogen by *Azotobacter* sp.

The Fe-protein is a homodimer that contains two nucleotide binding sites (MgATP or MgADP), one on each subunit and a single 4Fe–4S cluster that bridges the two subunits (Figure 3). The MoFe-nitrogenase is a α, β, heterotetramer. Each α, β dimeric unit contains two unique metalloclusters: a P-cluster (8Fe–7S) and a FeMo-cofactor (FeMo-co). During the catalytic cycle, a Fe-protein binds to one MoFe-protein αβ unit. During this encounter, one electron is transferred from the cluster 4Fe–4S to the MoFe protein. This electron transfer step is coupled to the hydrolysis of a minimum of two MgATP molecules. Following electron transfer and ATP hydrolysis, the Fe protein disengages from the MoFe protein and a new Fe protein binds in its place to repeat the cycle (Figure 3). Given that only one electron is transferred per cycle, a minimum of eight encounters must occur to reduce N as demonstrated by the Eq. 1. Detailed descriptions of the Fe-protein, P-clusters, and cofactor are available in Walker (2011). The major and minor clusters of genes encoding the nitrogenase complex enzymes were extensively studied in *A. vinelandii*. Detailed descriptions of the genome sequence of *A. vinelandii* and *nif* genes were reported by Setubal et al. (2009) and Poza-Carrión et al. (2015).

This is an energetically demanding process, requiring chemical energy in the form of phosphodiester bond energy from ATP (Setubal et al., 2009; Hamilton et al., 2011; Ledbetter et al., 2017; Segal et al., 2017; Batista Bueno and Dixon, 2019). Nevertheless, this process remains less demanding in energy and environmentally friendly compared to industrial N fixation through the energy-extensive Haber–Bosch method or geochemical processes, such as lightning (Gruber and Galloway, 2008; Thamdrup, 2012; Connor and Holland, 2017).

In addition, *Azotobacter* species could be considered as an evolving bacterium, because in addition to using the classic Mo-containing enzyme for BNF, *Azotobacter* species are able to synthetize one or more alternative nitrogenases under conditions where there is a lack of molybdenum. *A. vinelandii* for example was found to encode three different nitrogenase enzymes with different structural sub-units: (1) the traditional Mo-nitrogenase (2) a Vanadium containing enzyme (nitrogenase-2, encoded by the *vnf-H*, *vnf-D*, *G, K* genes), and (3) an iron containing nitrogenase (nitrogenase-3, encoded by the and *H*, *D*, *G*, *K* gene cluster) (Robson et al., 1986; Leigh, 2002).

Understanding the mechanisms employed by *Azotobacter* species to accomplish aerobic BNF could be integral to determining how to transfer this process into the aboveground bacteria, tissues of land plants, or better utilize this process in leaf endophytes or other endophytes associated with aboveground photosynthetic tissues in plants (Barney, 2020).

### Phosphorus Solubilization by *Azotobacter*

Besides N, P is a major nutrient, which plays an important role in plant physiology and biochemistry as well as in microorganism physiology such as BNF. Soils usually contain large amounts of total P in different available forms, including insoluble forms such as tri-calcium P (Ca_3_PO_4_)_2_, aluminum P (Al_3_PO_4_), and iron P (Fe_3_PO_4_). Unfortunately, compared to the other major nutrients, P is by far the least mobile and available nutrient to plants in most soils, even if the total soil P content is well beyond plant needs (400–1.200 mg/kg) (Nosrati et al., 2014). The poor mobility of soil P is due to the large reactivity of P ions with numerous soil constituents (Hinsinger, 2001), with only a small fraction of small P is available for plant growth (<1 mg P Kg^–1^) (Rodrìguez and Fraga, 1999; Richardson et al., 2009; Barker et al., 2015).

However, these forms may be converted to soluble P by soil phosphate-solubilizing microorganisms (PSMs) (Gupta et al., 2007; Song et al., 2008; Khan et al., 2013; Sharma et al., 2013; Kumawat et al., 2017). Numerous soil microflora were reported to solubilize insoluble P complexes into soluble forms readily absorbed by plants (Sashidhar and Podile, 2010). Among the phosphate-solubilizing bacteria (PSB), *Bacillus* and *Pseudomonas* are the most common along with some *Azotobacter* species also known for their P solubilizing capacity. A study by Hafez et al. (2016) demonstrated that *A. vinelandii* strain was able to solubilize up to 43% of the Abu Tartur phosphate rock in Egypt, while another study by Yi et al. (2008) showed that *Azotobacter* exopolysaccharides (EPS) were the main factor in the microbial solubilization of tricalcium P (TCP). *Azotobacter* species were also found to improve their P solubilizing through mutagenesis starting from soil isolates (Kumar et al., 2001). Therefore, these microorganisms are used as biofertilizers in order to compensate or even increase benefit of chemical fertilizers (Narula et al., 2000; Kumar et al., 2001; Nosrati et al., 2014).

The solubilization of insoluble P mechanism remains a research subject (Illmer and Schinner, 1995; Khan et al., 2007; Buch et al., 2008). Solubilization of P through low molecular weight organic acids has been a well-studied and a widely accepted theory being the main solubilization mechanism, and various studies have identified and quantified organic acids and defined their role in solubilization (Maliha et al., 2004; Khan et al., 2010; Marciano Marra et al., 2012; Azaroual et al., 2020). This process involves the acidification of microbial cells and their surroundings, leading to the release of P-ions from the P-mineral by H^+^ substitution for calcium (Trivedi and Sa, 2008). However, the efficiency of P solubilization process depends on the type and the amount of organic acids released, with assumption that the quality of the organic acid released is more important than the total amount of acids (Scervino et al., 2010). Other studies suggested that P solubilization can be done by other mechanisms besides the release of organic acids (Asea et al., 1988; Illmer and Schinner, 1992; Chen et al., 2006).

Another important aspect is the relation between BNF rates and soil nutrients availability (especially P). It is well established that BNF is often limited by the low P availability in soils, however Mills et al. (2004) found no exclusive P limitation at any of their experimental sites where the BNF seems to be limited by Mo alone in P rich soils and co-limited by both Molybdenum and P in P poor soils. Another study suggested that the BNF limitation by P and Mo is a dynamic process. P can likely limit BNF in the early stage of the growing season, while Mo is limiting factor in mid-season (Jean et al., 2013).

### *Azotobacter* Tolerance to Stress

In the soil ecosystem, populations of *Azotobacter* sp. are affected by soil physicochemical parameters such as organic matter, pH, temperature, soil depth, soil moisture, and soil salinity (Kizilkaya, 2009). The NaCl concentrations affected the PGPR activities of *Azotobacter*, mainly BNF in soil. However, some species of *Azotobacter* are known to tolerate salt concentrations of up to 10% NaCl. e.g., *Azotobacter salinestris* was shown to tolerate 8% NaCl concentration, but the total CFU/mL values were reduced compared to lower NaCl concentrations. In response to temperature, *Azotobacter* is a typical mesophilic organism which thrives at optimum temperatures of 25–30°C for growth and physiological properties. The minimum temperature for the growth of *Azotobacter* evidently lies on little above 0°C. *Azotobacter* cells cannot tolerate high temperatures, although they can survive at 45–48°C by forming cysts which germinate under favorable conditions (Saribay, 2003). *A. salinestris* survived at 45°C and recorded an optimum growth rate at 35°C, the growth reduced with increasing temperatures above 35°C.

The presence of *Azotobacter* populations in soil ecosystems is controlled by pH. Generally, lower pH (<6.0) decreases *Azotobacter* population and in some cases, completely inhibits their growth. Acidic soils have unfavorable properties of poor and physiologically active nutrients and unsatisfactory air–water regime, so that the presence of *Azotobacter* in these soils was very low or even absent (Andjelković et al., 2018). An optimum pH of 7–7.5 is favorable for the physiological functions of *Azotobacter*. At this pH, population number may fall between 10^2^ and 10^4^ per gram of soil (Becking, 2006). Meanwhile, *A. chroococcum* survived at pH 9 and its growth was not inhibited at higher pH values, whereas *A. salinestris* was sensitive to pH above 9 and no growth was observed above this range.

### *Azotobacter* Cysts Confer Unique Tolerance Traits and Survival Abilities

*Azotobacter* species possess some unique features such as cysts formation (Sadoff, 1975). The formation of cysts is induced naturally in face of unfavorable and extreme conditions such as high or low temperatures, freezing, salinity, and drought. The cyst formation is induced also in response to changes in nutrients concentrations in the medium or the addition of some organic substances such as ethanol, n-butan-1-ol, or β-hydroxybutyrate. It is also affected by aldehyde dehydrogenase and the response regulator AlgR (Núñez et al., 1999). These morphological changes are accompanied by metabolic shifts, changes in catabolism, respiration and biosynthesis of macromolecules. Cysts of *Azotobacter* are spherical and consist of the so-called “central body,” a reduced copy of vegetative cells with several vacuoles and a “two-layer shell.” The inner part of the shell has a fibrous structure called intine, while the outer part has a hexagonal crystalline structure called exine. Numerous polyhydroxybutyrate granules are always observed within the central body, alginate is a major component of the capsule, and alkylresorcinols (a phenolic lipid) and alkylpyrones that are synthesized upon encystment induction replace the phospholipids of the cyst membranes and are components of the exine (Segura et al., 2014; Lara-López and Geiger, 2017). Some studies clearly indicated the role of small RNAs and LEA (Late embryogenesis abundant) proteins in the formation and resistance to desiccation and abiotic stresses in *Azotobacter* cysts (Castañeda et al., 2016; Rodriguez-Salazar et al., 2017). One of the main features of the cyst is its ability to withstand desiccation, being able to survive in dry soil for more than 10 years whereas vegetative cells stored under the same conditions were inactivated in less than 2 years (Vela, 1974). In particular, they are twice as resistant to UV light. They are also resistant to drying, ultrasound and gamma and solar irradiation, but not to heating (Wyss et al., 1961).

Encystment of *Azotobacter* strains in laboratory conditions can be induced upon induction of vegetative cells with specific reagents such as ethanol, n-butan-1-ol, or β-hydroxybutyrate. This process may be of great interest in *Azotobacter* bioformulation, mainly when fertilizer and phosphate rock are used in combination with the inoculant. It was shown that cyst formation at large-scale in biofertilizer product development using *Sinorhizobium meliloti*, *Azospirillum brasilense*, and *Azospirillum lipoferum*, extended the product shelf-life while maintaining its effectiveness (József et al., 2007). However, further studies are needed to investigate the behavior of cysts in natural soil conditions, since this feature makes *Azotobacter* species more resistant to soil and environment conditions and predators.

### Growth Promoting Traits and Other Substances Produced by *Azotobacter*

Besides BNF, the beneficial effects of *Azotobacter* on plant growth are also attributed to an improvement in root development, an increase in the rate of mineral uptake by roots as well as their antagonism against fungi and plant pathogenic bacteria. *Azotobacter* synthetizes and secretes considerable amounts of biologically active substances like B vitamins, nicotinic acid, pantothenic acid, biotin, heteroxins, and gibberellin, which enhance root growth of plants (Azcón and Barea, 1975; Patil et al., 2020). Inorganic and organic P solubilization by *Azotobacter* strains is another growth promoting trait which is characterized to screen efficient free-living N_2_-fixing bacteria (Narula et al., 2000; Nosrati et al., 2014; Hafez et al., 2016).

Another plant growth promoting trait showed by *Azotobacter* species is auxin (IAA) production. It is a fundamental phytohormone that modulates plant growth and development (Halliday et al., 2009; Grossmann, 2010). This phytohormone helps the production of longer roots and increases number of root hairs and lateral roots which are involved in nutrient uptake (Datta and Basu, 2000). It plays a central role in cell division, elongation, fruit development and senescence. Auxin initiates roots, leaves, and flowers (Phillips et al., 2011). Several works proposed that *Azotobacter* species can facilitate plant growth via synthetizing this phytohormone rather than N fixation (Behl et al., 2007; Ahmed and Holmström, 2014).

In addition to the production of these substances, some strains of *Azotobacter* (such as *Atropicalis tropicalis*, *Azorhizophilus paspali*, and *A. vinelandii*) have been characterized by their capacity to synthesize antifungal substances that inhibit the development of some phytopathogenic species such as *Helminthosporium* sp., *Macrophomina* sp., and *Fusarium* sp. (Bjelić et al., 2015). El_Komy et al. (2020) demonstrated that the use of a mixture of *Azotobacter*, *Azospirillum*, and *Klebsiella* significantly reduced the mycelial growth of certain pathogenic fungi such as *Macrophomina phaseolina*, *Rhizoctonia solani*, and *Fusarium solani*. Also, isolates of *A. vinelandii* have been characterized to have the ability to produce polysaccharides, such as alginate, at rates ranging from 4.88 to 5.26 g/L. Hydrogen cyanide (HCN) and siderophores production has been also characterized for *Azotobacter* species (Baars et al., 2015).

Potassium (K) and zinc (Zn) solubilization are part of the important potentials of how *Azotobacter* can promote plant growth. Wu et al. (2006) demonstrated the ability of the soil bacteria *A. chroococcum* to increase the bioavailability of Zn in the soil system. Various mechanisms are involved in this process, including the acidification. These microbes produce organic acids in soil which sequester the Zn cations and decrease the nearby soil pH (Alexander, 1997; Aung et al., 2020). Other mechanisms possibly involved in Zn solubilization include production of new siderophores family by *A. chroococcum* e.g., vibrioferrin, amphibactins, and crochelins which can bind iron in a hexadentate fashion using a new iron-chelating γ-amino acid. Such siderophores help bacteria to access iron resources but contribute also to control plant pathogens in the soil (Saravanan et al., 2011; Baars et al., 2018).

The capacity of Azotobacter species to solubilize K has been proven through several works (Singh et al., 2010; Sangeeth et al., 2012; Archana et al., 2013; Diep and Hieu, 2013). Other works suggested that *Azotobacter* species can not only solubilize K but also they can play an important role in improving K assimilation by plant (Wu et al., 2005; Singh et al., 2010).

Enzyme 1-aminocyclopropane1-carboxylate (ACC) deaminase is also a key trait produced by *Azotobacter* (Omer et al., 2016). ACC deaminase-producing organisms decrease plant ethylene levels which, when present in high concentrations, can lead to plant growth inhibition or even death (Honma and Shimomura, 1978; Glick et al., 2007). This enzyme is responsible for the cleavage of the plant ethylene precursor, ACC, into ammonia and -ketobutyrate by decreasing ACC levels in plants.

Many *Azotobacter* strains produce pigments that are involved in the metabolism of other microorganisms. For example, *A. chroococcum* forms dark-brown water-soluble pigment melanin which occurs at high levels of metabolism during BNF. This process is thought to protect the nitrogenase system from oxygen. Shivprasad and Page (1989) quantified the effect of *Azotobacter* on the overall microbial activity of the soil via the determination of soil dehydrogenase activity, which is an indication of the intensity of metabolic activity of microorganisms. In this research, dehydrogenase activity increased in all the variants where *Azotobacter* was applied.

## Nutrient Use Efficiency May Be Enhanced in Response to *Azotobacter* Inoculation

The importance of *Azotobacter* as microbial inoculant is convincingly established throughout various experiments and large number of field trials. Ritika and Utpal (2014) showed in their review that the use of *Azotobacter* as N-biofertilizer increased the growth and yield of various crops under field conditions with a percentage increase of up to 40% for Cauliflower and 15–20% for Maize compared to conventional fertilizers. These beneficial effects can be attributed to the biosynthesis of biologically active substances, the stimulation of rhizospheric microorganisms, the production of phytopathogenic inhibitors and improved nutrient availability of N, P, carbon, and sulfur, through BNF and mineralization of organic residues in soil (Lévai et al., 2008; Lenart, 2012).

Numerous studies described crop responses to *Azotobacter* inoculation under greenhouse and field conditions. Plant responses ranged from increase in seed germination rates, root development, enhancement in nutrient uptake, root and shoot biomasses and leaf number and area (Wani et al., 2016). Quality attributes such as protein content, fruit total soluble solids and fruit stability after harvest have also been reported. Other studies also demonstrated that using *Azotobacter* species either alone as biofertilizer or in combination with other beneficial species like PSB and *Azosprillum* improved crop yield and quality of different crops. Table 1 summarizes the effect of *Azotobacter* based biofertilizers on yield and quality improvement of different crops and conditions. High percentage increases in both yield and quality attributes are reported in Table 1.

**TABLE 1:** Effect of *Azotobacter* based biofertilizers on yields and quality improvement of different crops.

*Azotobacter* based Biofertilizers	Crops	Experimental design	Yield			Quality attributes			References
			B−	B+	% increase	B−	B+	% increase	
*Rhizobium* + *Azotobacter* + PSB + AMF (*mycorrhizal* fungi)	Cluster Bean	Field experiment in India	4.28 (t/ha)	4.99 (t/ha)	16.59				Deshmukh et al., 2014
*Azosprillum* + *Azotobacter* + PSB	Potato	Two filed experiments in Egypt	10.8 (t/ha)	17.6 (t/ha)	62.32	4.20 (% weight loss 60DAH)^a^	1.4 (% weight loss 60DAH)	66	El-sayed et al., 2014
*Azotobacter* + PSB	Capsicum	Field experiments in India	7.13 (t/ha)	9.27 (t/ha)	30.01	19.26 (Ascorbic acid mg/100 g)	21.20 (Vitamin C mg/100 g)	31	Jaipaul et al., 2011
*Azotobacter* + PSB + *Azosprillum*	Okra	Field experiments in College of Agriculture in India	448.03 (q/ha)	469.28 (q/ha)	4.74	172.96 (single fruit weight g)	183.53 (single fruit weight g)	6.14	Mal et al., 2014
*Azotobacter*	Cucumber	Greenhouse experiment in Iraq	4387.2 (kg/greenhouse)	5343.4 (kg/greenhouse)	21.7	87.0 (Fruit Size in cm)	92.7 (Fruit Size in cm)	6.5	Saeed et al., 2015
*Azotobacter*	Cabbage	Field experiment in India	33.47 (t/ha)	37.80 (t/ha)	12.9	13.91 cm (Head diameter)	15.55 cm (Head diameter)	11.79	Sarkar et al., 2010
*Azotobacter*	Cotton	Glass house experiments in Columbia	220 (g/plant)	250 (g/plant)	13.6	–	–	–	Romero-Perdomo et al., 2017
*Azotobacter* + PSB	Chickpea	Pot and field experiments	1469.9 (Kg/ha)	1991.4 (Kg/ha)	35.5	0.34 (g Fruit weight)	0.4 (g Fruit weight)	17.64	Ansari et al., 2015
*Azotobacter* + *Azosprillum*	Canola	Foliar application in field study	38048 (kg/ha)	38628 (kg/ha)	1.52	486 (kg/ha Protein yield)	516 (kg/ha protein yield)	6.17	Ahmadi-Rad et al., 2016
*Azotobacter* + *Glomus intraradices*	Safflower	Field study in Iran	33.43 g (weight of 1000 grains)	34.31 g (weight of 1000 grains)	2.63	226.4 kg/ha (Oil Yield)	277.5 kg/ha (Oil Yield)	22.5	Mirzakhani et al., 2014
*Azotobacter* + *Chlorella* + Nostoc	Rice	*In situ* assay	13 cm (Length of rice plant sprouts)	16.5 cm (Length of rice plant sprouts)	26.92	–	–	–	Zayadan et al., 2014
*Azotobacter* + PSB	Broccoli	Pot study	1.10 kg/plant (Weight of the curd)	1.29 kg/plant (Weight of the curd)	17.27				Singh et al., 2014
*Azotobacter* + PSB	Tomato	Field study in the experimental farm of Horticultural Research Station Kandaghat, India	659.14 q/ha	816.61 q/ha	23.8	4.33 °Brix (TSS^b^)	4.80 °Brix (TSS)	10.85	Singh et al., 2015b
*Azotobacter* + PSB	Carrot	Field experiment in India	14.6 t/ha	19.6 t/ha	34.24	10.3 °Brix (TSS)	12.3 °Brix (TSS)	19.42	Sarma et al., 2015
*Azotobacter*	Wheat	Field conditions in Serbia	2333 kg/ha	2667 kg/ha	14.32	89 % (wheat seed viability)	91 % (wheat seed viability)	2.25	Milošević et al., 2012

*^a^Weight loss percentage of potato tubers 60 Days After Harvest (DAH) stored at 10°C and 90% relative humidity (El-sayed et al., 2014). ^b^Total soluble solids expressed as Brix (Singh et al., 2015b).*

## *Azotobacter* Species and Improved Tolerance of Plants to Biotic and Abiotic Stresses

Drought and salinity are among the major environmental constraints that limit growth, productivity, and quality of crops (Yang et al., 2009). Screening of various salt-tolerant strains of *Azotobacter* has revealed that some strains are able to colonize the rhizosphere successfully and promote plant growth under stress conditions. Multiple facets of *Azotobacter* mechanisms could explain their plant stress alleviation and may include additional properties beyond their characterized function of nitrogen fixation. All these properties could enhance the tolerance to abiotic and biotic stress in inoculated plants (Ruzzi and Aroca, 2015).

*Azotobacter* strains were found to enhance growth when applied with wheat under salt stress (Chaudhary et al., 2013). Additionally, inoculation of maize plants with *Azotobacter* has been reported to improve growth in saline stress conditions by improving sodium exclusion and potassium uptake (Rojas-Tapias et al., 2012; Latef et al., 2020). Moreover, *Azotobacter* species can protect several plants from biotic stress caused by plants’ pathogens. This capacity depends on their competition with the indigenous microbial and fungal strains and their colonization ability in the soil and rhizosphere (Goel et al., 1997). HCN and siderophores production agents (Ponmurugan et al., 2012) and the production of antimicrobial compounds such as 2,3-hydroxybenzoic acid, aminochelin, azotochelin, protochelin, and azotobactin are also known to inhibit the growth of many common plant pathogens such as *Curvularia*, *Aspergillus*, *Fusarium*, and *Rhizoctonia* species (Bhosale et al., 2013).

Several works on drought stress tolerance using *Azotobacter* species as a solution demonstrated the efficacy of their use (Creus et al., 2004; Shirinbayan et al., 2019). Sandhya et al. (2009) noted an increase of resistance to water stress in sunflower plants treated with EPS produced by *Azotobacter*, probably due to their ability to improve soil structure in the rhizosphere. The EPS produced by *Azotobacter* are essential molecules to maintain cellular hydration and biofilm formation under desiccating conditions. The polysaccharides are able to form various structures within a biofilm and may interact with a wide range of other molecules, including lectins, proteins, and lipids (Chang et al., 2007). They also revealed a high adsorption rate of metals (Gauri et al., 2011). EPS of *Azotobacter* directly bind and uptake heavy metals like Cd and Cr in the contaminated soils (Joshi and Juwarkar, 2009).

## Genetic Engineering of *Azotobacter*

For the large-scale production of *Azotobacter*, it is necessary to take into consideration the improvement of some cultural and nutritional parameters in order to improve its growth in fermentation process avoiding contamination, as well as to improve its capacity as biofertilizer (Gomare et al., 2013).

There are various methods that can be followed to enhance *Azotobacter* species capabilities with the help of genome engineering and synthetic biology for either addition or deletion of targeted gene(s).

For *Azotobacter* nitrogen fixation, previous evidence suggested that *nif-A* acted as an activator of nitrogenase expression, while *nif-L* acted as an anti-activator (Little et al., 2006). With the presence of oxygen or ammonium, *nif-L* interacts with the *nif-A* and blocks its function (Das, 2019).

Several works have considered these properties to increase the level of ammonium released. Bali et al. (1992) were able to obtain high levels of release compared to wild strains by only disrupting a section of the *nif-L* gene and leaving *nif-A* intact. While Ortiz-Marquez et al. (2012) constructed an almost complete suppression of *nif-L*, resulting in a strain that also accumulated significant ammonium levels in the medium compared to unmodified strains. Bageshwar et al. (2017) deleted a part of the negative regulatory gene *nif-L* in *A. chroococcum* strain named HKD15 that was able to enhance wheat yield with 60% and reduce urea fertilizer.

Besides increasing *Azotobacter* nitrogen fixing capacity, improving other capacities of this species will be useful, such as generating strains that produce alginates with specific chemical characteristics (Galindo et al., 2007) or engineering their capacities to solubilize phosphate (Sashidhar and Podile, 2009).

Genetic engineering of *Azotobacter* species could be also adopted in the formulation processes, in order to improve encystment capacity and produce high resilient cysts with longer shelf life and resistance to contamination and harsh environment conditions. Such applications are still under-studied and need more scientific efforts in order to develop new generation of *Azotobacter* based inoculant.

## Exploiting *Azotobacter* as a Candidate Bacterium for Biofertilizers

*Azotobacter* has been known for their beneficial effects on crop growth and yield through BNF, biosynthesis of biologically active substances, stimulation of rhizospheric microbes and production of phyopathogenic inhibitors (Lenart, 2012). This kind of bacteria is capable of surviving under severe conditions of temperature and water availability by converting to a more resistant form than the vegetative cells (Sadoff, 1975). All these capacities give them a possibility to be applied as basis for biofertilizer products that can decrease the excessive use of chemical fertilizers.

The beneficial effects of *Azotobacter* and *Azospirillum* interaction on plants are mainly attributed to their capacity to improve root development, water and mineral uptake by roots, the displacement of fungi and plant pathogenic bacteria and to the BNF (El-Mokadem et al., 1989; Okon and Itzigsohn, 1995). Similarly, combined inoculation of *Azotobacter* and *Rhizobium* spp. has revealed a positive synergistic action resulting in significant increase in nodulation, increasing N content within roots and shoots of respiring/metabolizing plant cells, improving conditions within the rhizosphere and enhancing synergistic interactions between the host and *Azotobacter* sp. (Yadav and Vashishat, 1991).

**FIGURE 4:**
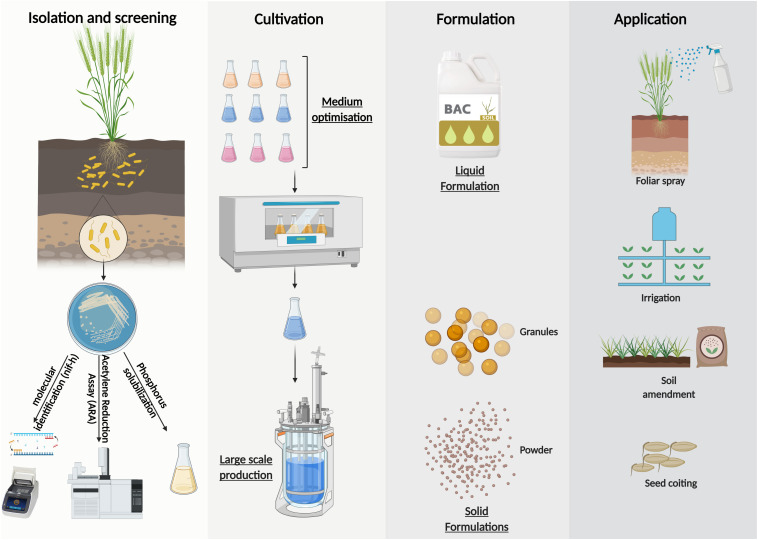
Formulation possibilities and large-scale production of inoculants from *Azotobacter* sp.

Large scale inoculants production of *Azotobacter* sp. could be summuriszed in 4 steps (Figure 4). It ranges from the isolation and screening of effective strains according to several characteristics including N fixation, P solubilization, etc. to mass production with a suitable culture medium and finally the choice of the formulation according to the application mode sought. Solid formulations may be subdivided into powders and granules depending on their particle sizes. In general, they are applied as seed coatings or soil amendments (Bashan et al., 2014). Whearas, liquid formulations are suitable for a wide range of application technologies, they may be coated directly onto the seed (with the use of adhesive) immediately prior to sowing (Bashan et al., 2014) or used as a coating for chemical fertilizers (Hindersah et al., 2020). They may also be delivered to the soil in−furrow during sowing or at a later stage via fertigation systems (Malusá et al., 2012). Furthermore, liquid formulations allows the treatment of above−ground plant parts, for example in form of a foliar spray (Jambhulkar et al., 2016).

## *Azotobacter*-Based Bioformulations (Biofertilizers) Market and IP Investments

The genus *Azotobacter* has been used as a biofertilizer more than a century (Gerlach and Vogel, 1902). Using *Azotobacter* strains as a bioinoculant for a wide range of crops (cereals, tomato, eggplant, carrot, and sugarcane), has been reported to lead to better yield results (Mrkovacki and Milic, 2001). N_2_-Fixing biofertilizers like *Rhizobium*, *Azotobacter*, and *Azospirillum*, which are majorly used for BNF in seed and soil treatment applications, currently represent the largest segment of the global biofertilizer market. Global N_2_-Fixing biofertilizers market was valued at USD 800 million in 2016 and is expected to reach USD 3 billion by the end of 2024 (Soumare et al., 2020), growing at a CAGR of about 14.3% during the forecast period. The global market for *Azotobacter*-based biofertilizer was valued at USD 212.2 million in 2017 and is expected to register a CAGR of 8.7% during the period 2020–2025 (Mordor intelligence market, 2020)^1^. Some biofertilizers based on *Azotobacter* available in the market are summarized in Table 2.

**TABLE 2:** Benchmark of *Azotobacter* based biofertilizers used around the globe (modified from Mishra and Arora, 2016).

Country	Company	Product	Bacteria	Crops
Southern and Eastern Russia	Natural resources	Azotobacterin	*Azotobacter chroococcum*	Field pea, soybean, chickpea, broad bean, narrow-leafed lupin, tomato, pepper, brinjal, sorrel, asparagus, estragon, etc.
	LLC EM Technology	Ekophit	*Azotobacter chroococcum*	
Australia	Mapleton Agri Biotec Pty Ltd	TwinN	*Azotobacter (soilborne species)*	Legumes and cereal crops
Canada	Nutri-Tech solutions	Nutri-Life Bio-P	*Azotobacter ssp.* and *Bacillus subtilis*	All crops
		Nutri-Life Bio-N	*Azotobacter ssp.*	All crops
India	T. Stanes & Company Limited	Symbion-N non associative type	*Azospirillum*, *Rhizobium*, *Acetobacter*, and *Azotobacter*	Sugar cane, sorghum, jowar, maize, cotton, tea and coffee
	Camson Bio Technologies Limited	CALZOTO	*Azotobacter sp.*	Legume crops, cereal crops, vegetable crops
	Gujarat State Fertilizers and Chemicals	Sardar Biofertilizers	*Azotobacter*, *Azospirillum*, and *PSB*	All types of crop
	Agri Life	Nitrofix AC	*Azotobacter chroococcum*	Large type of crops
		Nitrofix AV	*Azotobacter vinelendii*	Large type of crops
	KN Biosciences	Azopower	*Azotobacter sp.*	Horticulture and fruit crops
Hungary	PhylazonitKft	Phylazonit-M	*Bacillus megaterium* and *Azotobacter chroococcum*	Rice, maize
Colombia		Dimargon1	*Azotobacter chroococcum*	Rice, cotton

**FIGURE 5:**
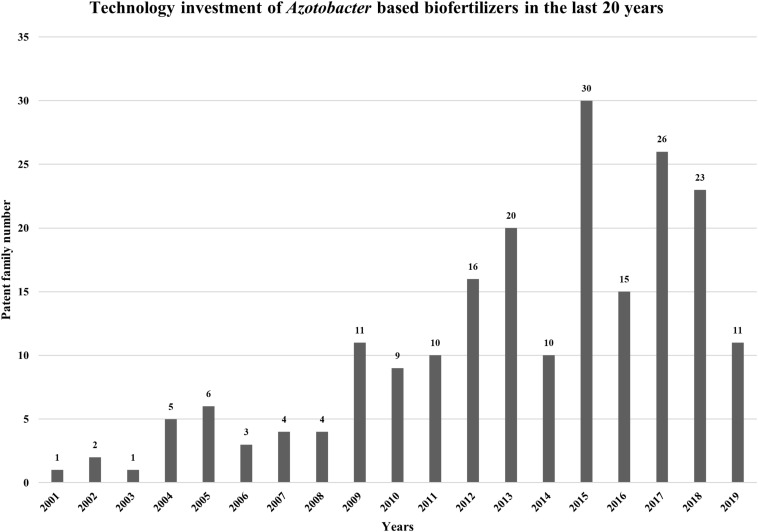
Technology investment and the evaluation dynamics of inventiveness of the studied of biofertilizers based on *Azotobacter* bacteria in the last 20 years.

In order to evaluate *Azotobacter* biofertilizers IP trends (patenting activity), an analysis was made using “IP Business Intelligence” of “Orbit Intelligence” services. The research was carried out using the following query: « *Azotobacter* and biofertilizer », which generated 233 IP patent documents with a corresponding statistical study (Figure 5).

The evaluation dynamics of inventiveness of the studied portfolio (Linear or exponential portfolio) indicates that the depositor is in the construction phase of its portfolio (more or less quickly). Contrariwise, a decrease in the number of patent families filed is generally symptomatic of a substantial decrease in R&D budgets and/or “intellectual property” budgets. Different profiles can be observed, and these profiles depend on the deposit strategy implemented by the depositor. Technology investment in the field of biofertilizers based on *Azotobacter* bacteria peaked in 2015, with 30 patent families as shown in Figure 5. This deposit peak can be explained by a massive deposit of actors at an instant t and in the opposite a hollow in patent deposit may be a result of repercussions of crisis or economic events on R&D budgets and consequently on patent filings. It should be noted that the last 2 years are incomplete, this is due to the publication period of 18 months between the filing of the application and its publication.

The results of our query showed that 17% of the resulting patent families belong to the top 10 IP players, besides that the International Patent Classification (IPC) has, in fact, been the subject of a grouping of work in 35 technological fields. “Basic materials chemistry” is predominant in the results, which belongs to IPC class of fertilizers and their manufacture (class C05). This class includes inorganic and organic fertilizers involving or not the addition of bacterial culture. Biotechnology is the second technological field that emerges from the statistical analysis of the patents research, which belongs to the IPC class of microbiology, enzymology, techniques of mutation, or genetic among others (class C12). Specifically, many patents resulting from the query are classified under the sub class C12N, which describes: micro-organisms or enzymes; compositions containing micro-organisms or enzymes; culture or preservation of micro-organisms, mutation or genetic techniques; culture media. Besides the distribution of these patenting activities, the extension strategies of the players in the sector were also studied. Thirty-nine patent families are applications filed at the European Patent Office and 23 are International patent applications [Patent Cooperation Treaty (PCT)]. The location of the extensions is a good indicator of the markets where players need to protect their invention in the location or region of interest. It should also be noted that some players protect the geographic areas where their competitors’ manufacturing sites are located (case of China and India).

The IP analysis showed that there is a need in investment in R&D in order to introduce new and innovative products. This analysis also identified the technological core of the actor in question. The least represented categories are future lines for identifying other potential applications of the actor’s patents.

## Concluding Remarks and Perspectives

It is clear that BNF can inexpensively supply an environmentally acceptable supplement for N resulting through the symbiotic and asymbiotic BNF either with legumes or other staple crops such as cereals. *Azotobacter* species are inevitably among the important contributors to BNF. Particularly, they are able to supply non-leguminous plants with significant amount of N, in addition to synthesizing plant growth promoting substances, which help increase availability of additional nutrients (P, K, and Zn) for better plant nutrition. Moreover, promising findings were highlighted regarding the ability of *Azotobacter* to be genetically modified in order to increase their capabilities to fix N_2_, to improve their colonization ability to plant, growth promotion traits and to improve their formulation effectiveness (Ambrosio et al., 2017; Bageshwar et al., 2017; Romero-Perdomo et al., 2017).

As per available knowledge gained so far, little is known about genes involved in plant-*Azotobacter* interactions and the key roles they likely perform. Further investigations, both basic and applied, are ultimately needed to find out whether BNF by *Azotobacter* species is a naturally occurring rhizosphere process that covers the bacteria need for N or a process induced in response to plant signals. Furthermore, the relationship between BNF rates and soil nutrients availability (especially P) needs to be unraveled. It is well established that BNF is limited by the P availability in soils, but the intervention and the limitation mechanism of BNF by P availability is not well discussed.

The compatibility of introduced *Azotobacter* species among the native microbes is still an unknown aspect to explore, but with the advance of omics technologies, there are opportunities to completely characterize and develop rhizosphere microbiome blueprints for individual crop species. This will help to understand changes in plant-rhizosphere microbiome composition and functions plausibly induced by *Azotobacter* either individually or in combination with other beneficial species. This will also improve the current understanding of how members of *Azotobacter* promote plant growth and nutrient use efficiency.

The response of crop to N-fertilizers is well understood, however, combination of microbial inoculants such as *Azotobacter* and N-fertilizers will require more investigations in order to determine whether the combined use of fertilizers and *Azotobacter* can ameliorate the BNF process or not. Another important aspect of required research is the production of *Azotobacter* microbial fertilizers, taking into consideration their ability to be transformed naturally to more resistant forms “cysts.” The induction of these forms could easily be integrated in fermentation processes, which produces a basic microbial bio-fertilizers materials. For this purpose, research must address several technological challenges such as the fermentation process, type of formulations, population of microorganisms and their release system. Thus, the development of a successful and environmentally friendly bioformulations should be made possible by combining interdisciplinary knowledge spanning microbiology and technological aspects. Promoting associative N_2_-Fixing *Azotobacter* for sustainable crops production and N nutrition has been an important biotechnological challenging interest. This is a growing and promising market and currently the focus should be on developing innovative and competitive *Azotobacter* based biofertilizers and this must go through a more substantial investment in R&D and IP.

## Author Contributions

All authors equally contributed to the preparation of the review, revised the text at different stages of the writing process, and read and approved the current manuscript.

## Conflict of Interest

YZ was employed by company Situation Innovation Group–OCP Group. The remaining authors declare that the research was conducted in the absence of any commercial or financial relationships that could be construed as a potential conflict of interest.

## Footnotes

1 Mordor intelligence Market (2020) https://www.mordorintelligence.com/industry-reports/azotobacter-based-biofertilizer-market [Accessed june, 2020].

## Abbreviations

SNF: symbiotic nitrogen fixation
N: nitrogen
N_2_: atmospheric nitrogen
BNF: biological nitrogen fixation
EPS: exopolysaccharide
PGPR: plant growth promoting rhizobacterium
P: phosphate
PSMs: phosphate-solubilizing microorganisms
PSB: phosphate-solubilizing bacteria
K: potassium
Zn: zinc
ACC: 1-aminocyclopropane1-carboxylate.
